# Supplementary material for: The Reinforced Spun Concrete Poles under Physical Salt Attack and Temperature: A Case Study of the Effectiveness of Chemical Admixtures
Source: Materials (Basel). 2020 Nov 12;13(22):5111. doi: 10.3390/ma13225111 (PMC7696209; doi:10.3390/ma13225111)
Supplement: Supplementary file 1 [file materials-13-05111-s001.pdf]

materials

MDPI

Supplementary Information

The Reinforced Spun Concrete Poles under Physical Salt Attack and Temperature: A Case Study of the Effectiveness of Chemical Admixtures

Romualdas Kliukas *, Arūnas Jaras and Ona Lukoševičienė

Department of Applied Mechanics, Faculty of Civil Engineering, Vilnius Gediminas Technical University, Saulėtekio al. 11, LT-10223 Vilnius, Lithuania; arunas.jaras@vgtu.lt (A.J.); ona.lukoseviciene@vgtu.lt (O.L.)
* Correspondence: romualdas.kliukas@vgtu.lt; Tel.: +37-05-2744858

Table S1. The main characteristics of the cross section and the results of testing the prismatic concrete control specimens under short-term axial compression cured under normal ambient conditions.

Type of Chem. Admixt.	Code of Prism Specimens	Wall Thickness of Specimens t (mm)	Prism Compr. Strength of Concrete f_c (MPa)	Average Strength of Concrete $\bar{f}_c$ (MPa)	Initial Modulus of Elasticity E_c (GPa)	Average Initial Modulus of Elasticity $\bar{E}_c$(GPa)
**The Control Prism Specimens after 25 Cycles of CWD**						
Admix. free	2196	85.5	46.1	49.1	31.1	29.0
	2198	83.5	52.0		26.9	
ACF-3M	2406	89.5	48.4	50.1	32.0	32.6
	2405	88.0	51.8		33.2	
Dofen	2076	89.0	63.9	62.8	37.4	36.2
	2077	90.5	61.7		35.0	
C-3	2281	93.0	65	66.8	34.3	36.5
	2282	95.0	68.5		38.6	
**The Control Prism Specimens after 50 Cycles of CWD**						
Admix. free	2215	76.0	58.8	55.6	29.1	31.8
	2218	77.5	52.4		34.5	
ACF-3M	2424	86.5	61.6	59.6	36.9	35.5
	2428	90.0	57.6		34.1	
Dofen	2068	95.0	78.6	77.10	41.7	41.0
	2066	94.5	75.6		40.3	
C-3	2256	94.5	71.4	70.0	37.4	38.0
	2254	95.0	68.6		38.5	
**The Control Prism Specimens after 75 Cycles of CWD**						
Admix. free	2121	88.0	58.8	57.1	35.1	33.6
	2124	89.0	55.4		32.1	
ACF-3M	2342	88.0	55.9	57.4	28.0	29.1
	2346	88.0	58.9		30.2	
Dofen	2014	89.0	68.7	66.2	34.8	37.0
	2018	90.0	63.7		39.2	
C-3	2243	92.0	69.5	67.6	36.4	35.7
	2247	88.0	65.7		35.0	

Table S2. The testing results of the prismatic specimens soaked in water and dried in the air at the temperature of 100 °C.

Type of Chem. Admixt.	Code of Prism Specimens	Wall Thickness of Specimens t (mm)	Prism Compr. Strength of Concrete f_c (MPa)	Average Strength of Concrete $\bar{f}_c$ (MPa)	Initial Modulus of Elasticity E_c (GPa)	Average Initial Modulus of Elasticity $\bar{E}_c$ (GPa)
**The Prism Specimens after 25 Cycles of CWD**						
Admix. free	2195	85.0	38.1	39.0	19.2	20.3
	2197	84.0	40.5		21.1	
	2191	87.0	38.,4		20.6	
ACF-3M	2408	93.0	40.1	43.0	26.4	24.6
	2401	93.0	48.4		22.1	
	2404	86.0	40.4		25.3	
Dofen	2078	88.5	53.4	56.8	25.8	27.2
	2075	83.5	58.5		26.3	
	2074	83.3	58.6		29.4	
C-3	2287	89.0	55.1	60.9	29.4	29.6
	2283	97.0	60.8		29.2	
	2288	90.0	66.7		30.1	
**The Prism Specimens after 50 Cycles of CWD**						
Admix. free	2217	76.0	40.8	40.0	22.0	20.0
	2216	75.0	40.6		20.4	
	2211	75.0	38.6		17.6	
ACF-3M	2427	82.5	53.9	54.4	26.4	26.6
	2426	80.5	52		24.7	
	2425	77.0	57.3		28.7	
Dofen	2067	86.0	70.2	68.4	30.4	28.8
	2065	85.0	65.6		28.0	
	2064	82.0	69.3		28.0	
C-3	2258	85.0	62.2	63.1	31.7	28.8
	2257	94.0	63		28.9	
	2255	89.5	64		25.9	
**The Prism Specimens after 75 Cycles of CWD**						
Admix. free	2122	88.5	37.0	38.8	18.0	19.5
	2125	89.0	38.6		21.0	
	2127	88.5	40.8		–	
ACF-3M	2341	88.0	46.3	44.2	18.8	19.8
	2344	88.0	43		20.4	
	2347	89.0	43.3		20.2	
Dofen	2011	90.0	58	55.6	25.1	27.8
	2013	88.5	55.2		29.0	
	2015	90.0	53.6		29.3	
C-3	2241	92.0	56.2	57.5	28.3	26.1
	2242	92.0	59.2		25.0	
	2245	91.0	57.1		25.1	

Table S3. The testing results of the prismatic specimens soaked in salt solutions and dried in the air at the temperature of 100 °C.

Type of Chem. Admixt.	Code of Prism Specimens	Wall Thickness of Specimens t (mm)	Prism Compr. Strength of Concrete f_c (MPa)	Average Strength of Concrete $\bar{f}_c$ (MPa)	Initial Modulus of Elasticity E_c (GPa)	Average Initial Modulus of Elasticity $\bar{E}_c$ (GPa)
**The Prism Specimens after 25 Cycles of CWD**						
Admix. free	2192	88.5	40.7	38.8	23.0	20.9
	2193	88.0	37.8		20.2	
	2194	88.0	37.9		19.5	
ACF-3M	2407	97.0	45.1	46.0	25.2	25.4
	2403	88.0	47.5		23.9	
	2402	90.0	45.3		27.2	
Dofen	2072	99.0	56.4	56.4	30.8	31.6
	2071	89.0	57.1		32.4	
	2073	80.5	55.6		-	
C-3	2284	95.0	63.6	61.3	28.3	30.5
	2285	92.0	61.4		32.7	
	2286	90.0	58.9		-	
**The Prism Specimens after 50 Cycles of CWD**						
Admix. free	2212	70.0	40.5	44.1	20.1	22.6
	2214	72.0	45.8		23.1	
	2213	69.5	46.0		24.6	
ACF-3M	2423	94.0	48.5	51.8	29.3	26.4
	2422	87.5	52.2		27.0	
	2421	77.0	54.7		22.9	
Dofen	2062	93.0	62.8	66.4	32.6	32.6
	2063	92.0	66.5		33.4	
	2061	80.0	69.8		31.8	
C-3	2251	92.0	57.8	60.8	32.3	31.9
	2253	85.0	64.0		32.9	
	2252	93.0	60.7		30.5	
**The Prism Specimens after 75 Cycles of CWD**						
Admix. free	2123	89.0	35.0	37.2	17.5	19.5
	2126	89.0	37.6		18.2	
	2128	89.5	39.0		22.8	
ACF-3M	2343	88.0	40.8	40.2	-	18.3
	2345	88.5	43.6		16.4	
	2348	89.0	36.2		202	
Dofen	2012	90.0	46.8	51.0	26.8	27.8
	2016	90.0	52		27.3	
	2017	91.0	54.2		29.3	
C-3	2244	91.0	52.8	52.7	29.8	27.5
	2246	90.0	51.3		28.0	
	2248	88.0	54.0		24.7	

Publisher's Note: MDPI stays neutral with regard to jurisdictional claims in published maps and institutional affiliations.

© 2020 by the authors. Licensee MDPI, Basel, Switzerland. This article is an open access article distributed under the terms and conditions of the Creative Commons Attribution (CC BY) license (http://creativecommons.org/licenses/by/4.0/).
